# Solidified Chloroform

**Published:** 1893-05

**Authors:** 


### Solidified Chloroform.

A new discovery is described in the *Berichte* which is likely to throw some light upon the vexed and important question of chloroform and its impurities. Professor Anschutz, of Bonn, in the course of certain researches in which the preparation of salicylic anhydride ($C_6H_4CO_2$) was involved, had occasion to use chloroform in the process, when he found that the mixed solution after being left for some time deposited in beautiful crystalline form a compound of chloroform with salicylic anhydride. A similar compound is formed also when ortho-cresotinic acid is substituted for the salicylide. The salicylide contains about 33 per cent of chloroform and the cresotinic compound about 30 per cent. Both bodies yield very pure chloroform when heated to 100° C.—a temperature considerably below their melting points. The cresotinic compound is, however, the more stable body, decomposing but little in the air, while the salicylide, under the same conditions, slowly gives off chloroform in a state of remarkable purity. Inasmuch as none of the usual impurities of chloroform crystallize along with these compounds, the process would appear to afford a method for the purification of chloroform on more satisfactory lines, for repeated crystallization is a method which yields, as every chemist knows, the purest and most refined products. Moreover, a solid chloroform compound is, as will be imagined less likely to undergo decomposition than a liquid compound, while the advantage of being able to transport chloroform practically in a solid form (for by simply warming the compound pure chloroform may be obtained) is one of obvious value. Meanwhile the results of clinical experiment with this new product will be awaited with eager interest—this being the test that alone can decide its value for anæsthetic purposes, however "chemically pure" the substance may be.—*Lancet.*

